# Evidence‐searching capability among health care professionals: a comparative study

**DOI:** 10.1186/s12909-021-02565-3

**Published:** 2021-02-25

**Authors:** Szu-Yu Chi, Yu-Shiun Tsai, Tien-Pei Fang, Tao-Hsin Tung, Ching-Chi Chi

**Affiliations:** 1 Department of Political Science, College of Social Sciences, National Taiwan University, 1, Sec 4, Roosevelt Rd, 10617 Taipei, Taiwan; 2 Department of Medical Education, Chang Gung Memorial Hospital, Chiayi, 6, Sec West, Chia-Pu Rd, 61363 Puzi, Chiayi Taiwan; 3 Department of Respiratory Therapy, Chang Gung Memorial Hospital, Chiayi, 6, Sec West, Chia-Pu Rd, 61363 Puzi, Chiayi Taiwan; 4 Department of Respiratory Care, Chang Gung University of Science and Technology, 2, Sec West, Chia-Pu Rd, 61363 Puzi, Chiayi Taiwan; 5 Evidence-Based Medicine Center, Taizhou Hospital of Zhejiang Province affiliated to Wenzhou Medical University, 150, Ximen Rd, 317000 Linhai, Taizhou, Zhejiang China; 6 Department of Dermatology, Chang Gung Memorial Hospital, Linkou, 5, Fuxing St, Guishan Dist, 33305 Taoyuan, Taiwan; 7 College of Medicine, Chang Gung University, 259, Wenhua 1st Rd, Guishan Dist, 33302 Taoyuan, Taiwan

**Keywords:** Evidence‐based medicine, Information storage and retrieval, Information seeking behavior, Knowledge acquisition

## Abstract

**Background:**

Evidence-based practice is among core competencies of health care professionals (HCPs). However, the levels of evidence-searching capability may differ among various disciplines of HCPs as they receive different education and trainings for various durations in medical schools and teaching hospitals.

**Methods:**

This study aimed to compare the evidence-searching capability among different disciplines of HCPs and identify which aspects need to be reinforced. From a teaching hospital, we recruited 80 HCPs of various disciplines and compared their evidence-searching capability by using a validated scale. To examine if sex and education levels affect evidence-searching capability, we performed a multiple linear regression analysis with collinearity diagnostics.

**Results:**

Physicians and pharmacists performed significantly better than other disciplines in the seven formative assessment items and the summative item (all *P* < 0.05). No collinearity was detected between discipline and age nor level of education. Except for the 2nd formative assessment item (correlation coefficient 0.24 ± 0.12, *P* = 0.04), participant’s levels of education did not affect evidence-searching capability. Age was associated with lower evidence-searching capability in five formative and the summative assessment items.

**Conclusions:**

We found a better evidence-searching capability among physicians and pharmacists than other HCPs who may require more training on evidence-searching skills. Also, evidence-searching skills training should be provided to HCPs irrespective of age and education levels.

## Background

Over the past few decades, evidence-based medicine (EBM) has become an important issue in clinical practice, medical education, and clinical research. Also, evidence-based practice has been listed by the US Institute of Medicine as one of five core competencies of health care professionals (HCPs). EBM aims to apply the best evidence in resolving clinical uncertainty and efficiently provide effective health care [1]. The practice of EBM is composed of five steps (i.e. ‘5A’): (1) asking a focused question; (2) acquiring relevant evidence; (3) appraisal of the evidence obtained; (4) applying the evidence to clinical care; and (5) auditing of the evidence-based performance [2]. However, in the real world, HCPs are usually too busy to have adequate time to complete the whole 5A process [3]. Only EBM researchers have enough ability and time in practicing 5A and producing the best evidence for clinical application. Therefore, the goal of EBM education is no longer expecting all HCPs to have the ability to go through the 5A process, but enabling them to acquire the best evidence efficiently [4, 5].

There are various disciplines of HCPs including physicians, registered nurses, pharmacists, allied health professionals such as respiratory therapists and dietitians. To maintain the normal operation of health care industry, administrative staff, clinical teachers, and research staff are also needed. They receive different education and trainings for various durations in medical schools and teaching hospitals. Also, the college entry requirements vary among different disciplines of HCPs, with physicians usually the strictest. Therefore, the levels of evidence-searching capability may differ among various disciplines of HCPs. The objective of this study was to compare the evidence-searching capability among different disciplines of HCPs and to identify which aspects need to be reinforced in EBM education.

## Methods

This study was a sub-analysis of a previous study in which a scale for measuring evidence-searching capability has been developed and validated [4]. From a teaching hospital, we recruited a total of 80 HCPs. We used a validated scale to measure the evidence-searching capability of these participants as shown in Table 1 [4]. This scale is composed of 15 items (numbered from F1 to F15) that assess different aspects of evidence-searching skills (formative assessment) and 1 summative rating item (numbered as S1) that assess the overall ability in evidence-searching (summative assessment). The first four formative assessment items (F1 to F4) examine the ability to build up the PICO structure (P standing for patient or population, I for intervention, C for comparison, and O for outcome) [6] of a clinical question and to propose associated search terms. The 5th to 15th formative assessment items (F5 to F15) test the ability in devising searching strategy and searching skills [4].

**Table 1:** Scale for measuring evidence-searching capability. Adapted from [4] with permission

No	Item
**F1**	Propose the search terms and synonyms for P (Patient/population)
**F2**	Propose the search terms and synonyms for I (Intervention)
**F3**	Propose the search terms and synonyms for C (Comparison)
**F4**	Propose the search terms and synonyms for O (Outcome)
**F5**	Identify and prioritise the use of appropriate secondary databases (e.g., the Cochrane Library, PubMed Clinical Queries)
**F6**	Use of both MeSH term and free text in searching databases
**F7**	Search the databases using the search terms for P (patient/population/participant) and I (intervention)
**F8**	Appropriate use of Boolean operators (AND, OR and NOT) in combining keywords/synonyms to create search strategy
**F9**	Ability to use the truncation function in searching databases
**F10**	Ability to use the MeSH function in *The Cochrane Library* to find synonyms
**F11**	Ability to use the dropdown menu in the Advanced Search webpage of *The Cochrane Library*
**F12**	Write down the number of ‘Reviews’ in ‘Cochrane Reviews’ in the search results of *The Cochrane Library*
**F13**	Ability to use the MeSH function in PubMed
**F14**	Ability to use PubMed Clinical Queries and obtain systematic reviews
**F15**	Ability to identify local publications
**S1**	Global rating score

### Statistical analysis

We compared the evidence-searching skills between different disciplines of HCPs and analyzed the 15 items and 1 summative rating item, respectively, by using the Kruskal-Wallis test with post-hoc Dunn’s test. To examine if age group and level of education affect evidence-searching capability, we performed a multiple linear regression analysis with collinearity diagnostics. Collinearity refers to near perfect linear combinations of two variables and multicollinearity involves more than two variables, leading to unstable regression estimates with high standard errors. We used variance inflation factor (VIF) to evaluate multicollinearity, with a VIF of > 10 indicating multicollinearity [7]. We considered HCPs aged > 35 years as having completed trainings and thus separated the participants’ age into two categories: junior HCPs (aged ≤ 35 years) and senior HCPs (aged > 35 years). The levels of education were separated into two levels: undergraduate degree (diploma or bachelor) and graduate degree (master or doctorate). A P value of < 0.05 was considered significant. The Stata 13.1 for Windows (StataCorp LP, College Station, US) was used for statistical analyses.

## Results

 The data on the evidence-searching capability of the 80 participants, including 23 men (28.8 %) and 57 women (71.3 %), were collected and analyzed. The disciplines and education levels of participants are presented in Table 2. Data on 2 administrative staff, 5 research assistants, and 2 clinical teachers were pooled to form an ‘Others’ group because the sample size was small (*n* = 9).

**Table 2:** Disciplines and education levels of participants

	Male participants (*n* = 23)	Female participants (*n* = 57)	Total (*n* = 80)
**Disciplines**			
** Physicians**	12 (52.2 %)	5 (8.8 %)	17 (21.3 %)
** Registered nurses**	0 (0.0 %)	23 (40.4 %)	23 (28.8 %)
** Pharmacists**	1 (4.3 %)	3 (5.3 %)	4 (5 %)
** Allied health professionals**	9 (39.1 %)	18 (31.6 %)	27 (33.8 %)
** Others**	1 (4.3 %)	8 (14.0 %)	9 (11.3 %)
**Education**			
** Undergraduate degree**	19 (82.6 %)	36 (63.2 %)	55 (68.8 %)
** Graduate degree**	4 (17.4 %)	21 (36.8 %)	25 (31.3 %)

The data on the 15 formative assessment items and 1 summative assessment item of participants are shown in Table 3. Various disciplines of HCPs significantly differed in the seven formative assessment items (F1 to F6 and F8) and the summative item (S1) (all *P* < 0.05). By using the post-hoc Dunn’s test, physicians and pharmacists did not differ in these item (all *P* > 0.05). However, both physicians and pharmacists differed from registered nurses, allied health professionals, and others in most of these items (*P* < 0.05, data not shown).

**Table 3:** Evidence-searching capability of various health care professionals

Item	Physicians (*n* = 17)	Nurses (*n* = 23)	Pharmacists (*n* = 4)	Allied health (*n* = 27)	Others (*n* = 9)	P^a^
**F1**	1.77 ± 0.31	1.09 ± 0.42	1.50 ± 0.41	1.35 ± 0.41	1.00 ± 0.56	**< 0.001**
**F2**	1.65 ± 0.34	0.98 ± 0.46	1.50 ± 0.41	1.17 ± 0.39	0.94 ± 0.63	**< 0.001**
**F3**	1.59 ± 0.32	1.26 ± 0.65	1.50 ± 0.00	0.78 ± 0.68	0.72 ± 0.87	**0.002**
**F4**	1.65 ± 0.49	0.98 ± 0.63	1.13 ± 0.75	0.85 ± 0.63	0.72 ± 0.75	**0.001**
**F5**	2.00 ± 0.00	1.94 ± 0.23	1.75 ± 0.29	1.96 ± 0.13	1.67 ± 0.71	**0.04**
**F6**	1.79 ± 0.25	1.39 ± 0.48	1.75 ± 0.29	1.61 ± 0.25	1.01 ± 0.58	**< 0.001**
**F7**	1.77 ± 0.31	1.46 ± 0.45	1.63 ± 0.48	1.63 ± 0.30	1.28 ± 0.67	0.10
**F8**	1.77 ± 0.40	1.22 ± 0.36	1.63 ± 0.25	1.43 ± 0.41	1.06 ± 0.39	**< 0.001**
**F9**	1.47 ± 0.77	1.50 ± 1.69	1.38 ± 0.95	1.46 ± 0.77	1.39 ± 0.74	0.99
**F10**	1.53 ± 0.87	1.44 ± 0.90	2.00 ± 0.00	1.63 ± 0.74	0.89 ± 1.05	0.18
**F11**	1.70 ± 0.59	1.52 ± 0.85	1.50 ± 1.00	1.26 ± 0.90	1.22 ± 0.97	0.46
**F12**	0.88 ± 0.93	1.00 ± 1.00	1.00 ± 1.15	1.30 ± 0.95	1.11 ± 1.05	0.68
**F13**	1.94 ± 0.24	1.48 ± 0.90	2.00 ± 0.00	1.82 ± 0.56	1.89 ± 0.33	0.26
**F14**	1.56 ± 0.50	1.09 ± 0.81	1.00 ± 1.15	1.52 ± 0.70	1.56 ± 0.63	0.23
**F15**	1.35 ± 0.49	1.02 ± 0.63	1.50 ± 0.00	1.24 ± 0.58	0.89 ± 0.70	0.18
**S1**	3.79 ± 0.61	2.54 ± 0.81	3.25 ± 0.87	3.04 ± 0.44	2.22 ± 0.83	**< 0.001**

^a^Kruskal-Wallis test

As shown in Table 4, the multiple linear regression analysis with collinearity diagnostics detected no collinearity among the age group, level of education, and discipline of participants (all VIF < 10). Except for the 2nd formative assessment item (correlation coefficient 0.24 ± 0.12, *P* = 0.04), participants’ levels of education did not significantly affect evidence-searching capability. Senior age (> 35 years) was negatively associated with evidence-searching capability in five formative assessment items (F1, F2, F7, F8, and F10) and the summative assessment (S1) item.

**Table 4:** Multiple linear regression analysis with collinearity diagnostics. For each coefficient, corresponding standard error was reported

Item	Registered nurses			Pharmacist			Allied health			Others			Age > 35			Graduate degree		
	Coefficient	P	VIF	Coefficient	P	VIF	Coefficient	P	VIF	Coefficient	P	VIF	Coefficient	P	VIF	Coefficient	P	VIF
**F1**	**-0.52 ± 0.16**	**0.001**	2.43	-0.31 ± 0.24	0.18	1.20	**-0.39 ± 0.13**	**0.003**	1.74	**-0.73 ± 0.17**	**< 0.001**	1.41	**-0.23 ± 0.24**	**0.03**	1.33	-0.05 ± 0.11	0.63	1.33
**F2**	**-0.68 ± 0.16**	**< 0.001**	2.43	-0.25 ± 0.24	0.29	1.20	**-0.50 ± 0.13**	**< 0.001**	1.74	**-0.75 ± 0.18**	**< 0.001**	1.41	**-0.24 ± 0.11**	**0.03**	1.33	**0.24 ± 0.12**	**0.04**	1.33
**F3**	-0.34 ± 0.24	0.17	2.43	-0.12 ± 0.36	0.74	1.20	**-0.82 ± 0.20**	**< 0.001**	1.74	**-0.88 ± 0.40**	**0.001**	1.41	-0.07 ± 0.17	0.69	1.33	0.08 ± 0.18	0.69	1.33
**F4**	-0.42 ± 0.24	0.08	2.16	-0.57 ± 0.35	0.10	1.20	**-0.76 ± 0.19**	**< 0.001**	1.74	**-0.86 ± 0.26**	**0.001**	1.41	-0.32 ± 0.16	0.05	1.33	-0.13 ± 0.17	0.45	1.33
**F5**	-0.15 ± 0.11	0.17	2.43	-0.28 ± 0.21	0.08	1.20	-0.06 ± 0.09	0.52	1.74	**-0.37 ± 0.12**	**0.002**	1.41	-0.01 ± 0.07	0.92	1.33	0.15 ± 0.08	0.06	1.33
**F6**	**-0.34 ± 0.14**	**0.02**	2.43	-0.10 ± 0.21	0.62	1.20	-0.18 ± 0.12	0.129	1.74	**-0.74 ± 0.16**	**< 0.001**	1.41	-0.19 ± 0.10	0.051	1.40	0.08 ± 0.10	0.47	1.33
**F7**	-0.25 ± 0.15	0.11	2.43	-0.21 ± 0.23	0.35	1.20	-0.14 ± 0.13	0.28	1.74	**-0.50 ± 0.17**	**0.004**	1.41	**-0.22 ± 0.11**	**0.04**	1.40	0.11 ± 0.11	0.34	1.33
**F8**	-0.20 ± 0.13	0.14	2.13	-0.18 ± 0.19	0.35	1.20	**-0.29 ± 0.11**	**0.01**	1.74	**-0.37 ± 0.09**	**< 0.001**	1.41	**-0.37 ± 0.09**	**< 0.001**	1.40	**-0.25 ± 0.10**	**0.01**	1.33
**F9**	0.15 ± 0.10	0.60	2.43	-0.03 ± 0.42	0.94	1.20	0.02 ± 0.23	0.93	1.74	-0.02 ± 0.31	0.96	1.41	0.06 ± 0.20	0.75	1.33	-0.26 ± 0.21	0.21	1.33
**F10**	-0.04 ± 0.31	0.90	2.43	0.30 ± 0.46	0.52	1.20	0.09 ± 0.25	0.74	1.74	**-0.69 ± 0.34**	**0.048**	1.41	**-0.47 ± 0.21**	**0.03**	1.33	0.34 ± 0.23	0.14	1.33
**F11**	-0.19 ± 0.31	0.57	2.43	-0.21 ± 0.48	0.70	1.20	-0.45 ± 0.27	0.10	1.74	-0.48 ± 0.36	0.18	1.41	0.00 ± 0.22	0.99	1.33	0.00 ± 0.24	1.00	1.33
**F12**	0.08 ± 0.38	0.84	2.43	0.13 ± 0.56	0.82	1.20	0.41 ± 0.31	0.19	1.74	0.22 ± 0.42	0.60	1.41	0.05 ± 0.26	0.84	1.33	0.02 ± 0.28	0.94	1.33
**F13**	-0.38 ± 0.24	0.11	2.43	0.01 ± 0.34	0.97	1.20	-0.12 ± 0.19	0.54	1.74	-0.04 ± 0.26	0.88	1.41	-0.18 ± 0.16	0.28	1.33	0.02 ± 0.17	0.91	1.33
**F14**	**-0.66 ± 0.27**	**0.02**	2.43	-0.63 ± 0.40	0.12	1.20	-0.08 ±0.22	0.71	1.74	-0.09 ± 0.30	0.75	1.41	-0.02 ± 0.19	0.91	1.33	0.34 ± 0.20	0.10	1.33
**F15**	**-0.51 ± 0.22**	**0.03**	2.43	0.16 ± 0.33	0.62	1.20	-0.14 ± 0.18	0.44	1.74	**-0.52 ± 0.24**	**0.04**	1.41	0.17 ± 0.15	0.26	1.33	0.14 ± 0.16	0.38	1.33
**S1**	**-1.04 ± 0.25**	**< 0.001**	2.43	-0.66 ± 0.36	0.07	1.20	**-0.74 ± 0.20**	**< 0.001**	1.74	**-1.54 ± 0.27**	**< 0.001**	1.41	**-0.44 ± 0.17**	**0.01**	1.33	0.05 ± 0.18	0.77	1.33

*VIF* Variance Inflation Factor

## Discussion

This study found a higher level of evidence-searching capability of physicians and pharmacists than other disciplines of HCPs, especially in the building up of search terms, prioritized use of secondary databases, use of both Medical Subject Headings (MeSH) term and free text in searching, and devising search strategy by using appropriate Boolean operators. Also, physicians and pharmacists had a better overall ability in evidence-searching than other HCPs. Therefore, more EBM trainings including evidence-searching capability in the aforementioned skills should be provided for HCPs, especially those disciplines other than physicians and pharmacists.

The better evidence-searching capability found in physicians and pharmacists may be related to the different levels of contact with latest evidence. When compared to other disciplines of HCPs, physicians and pharmacists are in greater demand for up-to-date evidence on clinical trials as newly developed drugs are springing up over the past several decades. Therefore, physicians and pharmacists may have more experiences in searching databases than other HCPs.

Compared to HCPs of younger age, senior HCPs should have possessed better evidence-searching capability because of having a better understanding of the context of the clinical PICO question. By contrast, one previous study suggested younger age as a facilitating factor for evidence-based practice [8]. Also, our study illustrated that senior HCPs had a slightly lower ability in certain items of evidence-searching capability. Similarly, HCPs with graduate degree should have received more training in graduate school and thus a better ability and more experiences in searching databases than those with undergraduate degree. One Taiwanese study has found better performance in EBM skills including literature search in HCPs with a master degree than those with an undergraduate degree [9]. Another Australian study found more frequent access to electronic evidence resources among HCPs who had received postgraduate education than those who had not [10]. Nevertheless, our study found only in the second formative assessment item (‘Propose the search terms and synonyms for I (Intervention)’) did HCPs with a graduate degree have a slight better performance than those without a graduate degree. Our findings suggest that evidence-searching skills training should be provided to HCPs irrespective of age and education levels.

There are several limitations in this study. First, this pilot study was a sub-analysis and the participants were recruited from a single teaching hospital. However, the HCPs received education and training from various medical schools and teaching hospitals. Therefore, our findings may represent a miniature of EBM education. Also, one previous nationwide survey of 605 physicians and 551 registered nurses in 61 hospitals across Taiwan found that physicians possessed better self-rated evidence-acquiring skills than registered nurses [11]. Second, the sample size of the pharmacist group was only 4 in the present study and might have led to underpowering (type II error). Nevertheless, we detected a higher evidence-searching capability in pharmacists than other HCPs. Third, the setting of this study was limited to one country. The findings that certain evidence-searching skills need to be reinforced in EBM curriculum may not be extrapolated to other countries; yet there has been a trend advocating training on evidence-acquiring ability at the point of care [5, 12].

## Conclusions

Our study suggested a higher level of evidence-searching capability among physicians and pharmacists than other HCPs. HCPs other than the two disciplines may require more training on evidence-searching skills, especially in the building up of search terms and strategy as well as prioritized use of appropriate secondary databases. Also, our study illustrated that senior age and graduate education did not increase evidence-searching capability. Therefore evidence-searching skills training should be provided to HCPs irrespective of age and education levels.

## Data Availability

The datasets used and/or analysed during the current study are available from the corresponding author upon reasonable request.

## Abbreviations

EBM: Evidence-based medicine
HCP: Health care professional
MeSH: Medical Subject Headings
VIF: Variance inflation factor

## References

[1] Sackett DL, Rosenberg WM, Gray JA, Haynes RB, Richardson WS (1996). Evidence based medicine: what it is and what it isn’t. BMJ.

[2] Chi CC (2013). Evidence-based dermatology. Dermatologica Sinica.

[3] Barzkar F, Baradaran HR, Koohpayehzadeh J (2018). Knowledge, attitudes and practice of physicians toward evidence-based medicine: A systematic review. J Evid Based Med.

[4] Tsai YS, Fang TP, Chi CC (2019). A scale for measuring evidence-searching capability: A development and validation study. J Eval Clin Pract.

[5] Hurwitz SR, Slawson DC (2010). Should we be teaching information management instead of evidence-based medicine?. Clin Orthop Relat Res.

[6] Centre for Evidence-Based Medicine. Asking Focused Questions. 2014. https://www.cebm.net/2014/06/asking-focused-questions/. Accessed 28 Jun 2020.

[7] Marcoulides KM, Raykov T (2019). Evaluation of variance inflation factors in regression models using latent variable modeling methods. Educ Psychol Meas.

[8] Li S, Cao M, Zhu X (2019). Evidence-based practice: Knowledge, attitudes, implementation, facilitators, and barriers among community nurses-systematic review. Medicine.

[9] Chang HC, Wu CL, Chao PC, Lin LY, Yu YT, Lin SJ (2014). An analysis of literature searching anxiety in evidence-based medicine education. J Edu Media Libr Sci.

[10] Jansen L, Rasekaba T, Presnell S, Holland AE (2012). Finding evidence to support practice in allied health: peers, experience, and the internet. J Allied Health.

[11] Chiu YW, Weng YH, Lo HL, Hsu CC, Shih YH, Kuo KN (2010). Comparison of evidence-based practice between physicians and nurses: a national survey of regional hospitals in Taiwan. J Contin Educ Health Prof.

[12] Slawson DC, Shaughnessy AF (2005). Teaching evidence-based medicine: should we be teaching information management instead?. Acad Med.

